# Extensive Cecal Angioectasia Causing Chronic Occult Gastrointestinal Blood Loss and Iron-Deficiency Anemia: A Case Report

**DOI:** 10.7759/cureus.111666

**Published:** 2026-06-28

**Authors:** Abeer Qasim, Priscilla Lajara, Pragathi Munnangi, Rayan Alataa, Elona Shehi

**Affiliations:** 1 Gastroenterology, BronxCare Health System, Bronx, USA; 2 Internal Medicine, BronxCare Health System, Bronx, USA

**Keywords:** cecal angiodysplasia, endoscopic coagulation, gastrointestinal angiodysplasia, iron deficiency anemia, lower gastrointestinal bleeding, recurrent gastrointestinal bleeding

## Abstract

Gastrointestinal angioectasia is considered one of the leading causes of lower gastrointestinal bleeding in elderly patients. It represents the most frequent vascular abnormality within the gastrointestinal tract, with lesions most commonly found on the right side of the colon. Endoscopic management includes several techniques aimed at removing abnormal mucosal lesions. However, recurrent bleeding after endoscopic therapy is common and may require repeated endoscopic interventions or additional therapeutic modalities. Patients typically present with intermittent, painless gastrointestinal bleeding. The lesions may be solitary or multiple, most frequently located in the stomach, small intestine, or right colon. Diagnosis is mainly established through endoscopy. Initial treatment usually involves endoscopic coagulation, while more severe cases may require angiographic embolization or surgical resection. We present the case of a 78-year-old man who presented with severe transfusion-requiring iron-deficiency anemia without ongoing overt gastrointestinal bleeding and was found to have an unusually large cecal angioectasia that differed from the small, flat lesions typically described in the literature. The lesion was successfully managed with mechanical clipping rather than conventional thermal ablation, highlighting an alternative endoscopic approach for selected large vascular lesions.

## Introduction

According to the American Gastroenterological Association (AGA), occult gastrointestinal (GI) bleeding refers to a positive fecal occult blood test, with or without iron deficiency anemia (IDA) [1]. Obscure GI bleeding is defined as recurrent or persistent bleeding from an unidentified source after normal gastroscopy, colonoscopy, and, per the 2007 update, small bowel imaging. It is further classified as "obscure overt" when visible bleeding is present and "obscure occult" when it is not [2].

Angiodysplasias, also referred to as arteriovenous malformations (AVMs), angiectasias, or vascular ectasias, can occur throughout the gastrointestinal tract, with prevalence increasing with age. They represent the most common vascular abnormalities of the GI tract and are recognized causes of both occult and obscure gastrointestinal bleeding [2]. Angioectasia lesions found incidentally during colonoscopy do not require treatment in patients without a history of GI bleeding or anemia. Treatment is indicated only when there is evidence of occult or overt bleeding. 

We present the case of a 78-year-old male patient who presented with severe transfusion-requiring IDA without ongoing overt GI bleeding and was found to have an unusually large cecal angioectasia that differed from the small, flat lesions typically described in the literature. The lesion was successfully managed with mechanical clipping rather than conventional thermal ablation, highlighting an alternative endoscopic approach for selected large vascular lesions.

## Case presentation

A 78-year-old male patient with a past medical history of diabetes, hypertension, chronic kidney disease, compensated liver cirrhosis, hypothyroidism, and congestive heart failure presented to the emergency department with complaints of chest tightness, shortness of breath, and bilateral lower extremity edema. He denied any abdominal pain, nausea, vomiting, melena, hematochezia, or hematemesis. He had no history of prior surgeries. His medications included amlodipine, carvedilol, empagliflozin, hydralazine, levothyroxine, losartan, metformin, and rosuvastatin. He denied any known drug allergies. There was no family history of GI malignancies or other significant conditions. Social history was notable for chronic alcohol use; he reported drinking three to four beers daily for the past several years, with his last drink about 20 days ago. He had never undergone any prior endoscopic evaluation.

Upon arrival at the emergency department, the patient was hemodynamically stable with a blood pressure of 151/70 mmHg but required 2 liters of oxygen via nasal cannula. Initial laboratory work revealed a hemoglobin level of 7 g/dL, with no evidence of overt GI bleeding. After receiving one unit of packed red blood cells, his hemoglobin improved to 8.3 g/dL. His mean corpuscular volume (MCV) was 90.5 fL, and his platelet count was 189 × 10⁹/L. Iron studies showed iron at 26 µg/dL (low), ferritin at 12.7 ng/mL (low), and transferrin saturation at 9% (low). Although the MCV remained within the normal range, the markedly reduced ferritin, serum iron, and transferrin saturation supported IDA. The normocytic indices were likely influenced by coexisting chronic kidney disease and chronic inflammatory conditions, resulting in acute-on-chronic anemia. His pro-brain natriuretic peptide (proBNP) and troponin levels were elevated, prompting initial treatment for congestive heart failure exacerbation. The laboratory test results are given in Table 1.

**Table 1:** Laboratory test results → indicates change in values during hospital course

Laboratory Test	Patient Value	Reference Range	Notes
Hemoglobin (Hgb)	7.0 → 8.3 g/dL (baseline 8–9 g/dL)	Male: 13.5–17.5 g/dL	Low
White Blood Cell (WBC) Count	6.0 ×10³/µL	4.0–11.0 ×10³/µL	Normal
Hematocrit (Hct)	24.0%	Male: 41–53%	Low
Mean Corpuscular Volume (MCV)	90.5 fL	80–100 fL	Normal
Platelets	189 k/µL	150–400 k/µL	Normal
Iron	26 µg/dL	65–175 µg/dL	Low
Ferritin	12.7 ng/mL	Male: 30–400 ng/mL	Low
Transferrin	226.1 mg/dL	200–360 mg/dL	Normal
Unsaturated Iron Binding Capacity (UIBC)	261 µg/dL	111–343 µg/dL	Normal
Transferrin Saturation (TSAT)	9%	20–50%	Low
Haptoglobin	169 mg/dL	30–200 mg/dL	Normal
Lactate Dehydrogenase (LDH)	199 U/L	140–280 U/L	Normal
Blood Urea Nitrogen (BUN)/Creatinine	29 / 2.7 mg/dL	BUN: 7–20 mg/dL; Creatinine: 0.7–1.3 mg/dL	Acute kidney injury on chronic kidney disease
Pro–B-type Natriuretic Peptide (Pro-BNP)	4583 pg/mL	<125 pg/mL (<75 years)	Elevated
Troponin	69 → 64 ng/L	<14 ng/L	Mildly elevated, downtrending
Thyroid-Stimulating Hormone (TSH)	4.4 µIU/mL	0.4–4.5 µIU/mL	Normal
Activated Partial Thromboplastin Time (aPTT)	34.1 sec	25–35 sec	Normal
Prothrombin Time (PT)	12.4 sec	11–13.5 sec	Normal
International Normalized Ratio (INR)	1.02	0.8–1.1	Normal

**Figure 1:**
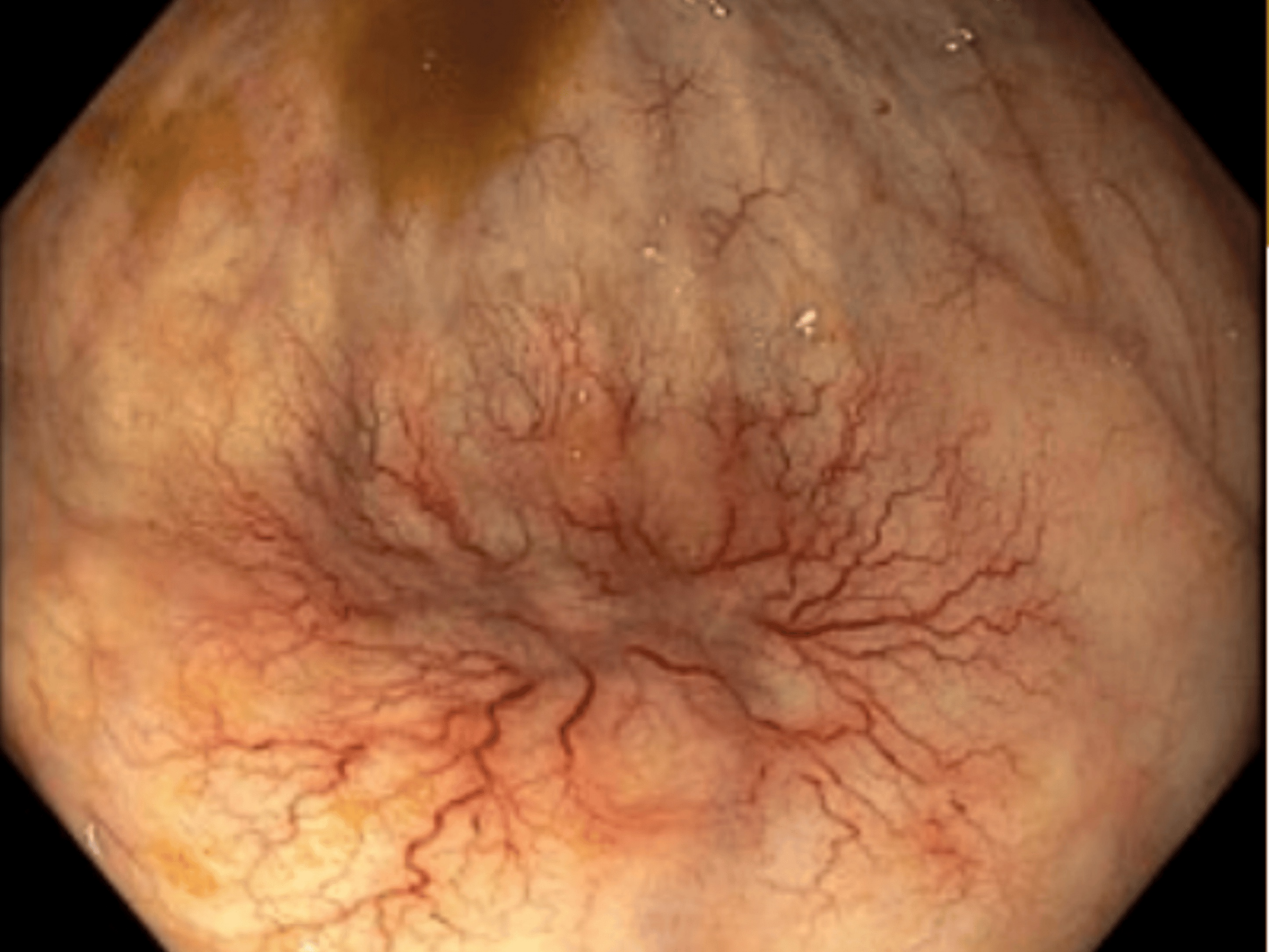
Colonoscopy demonstrating colonic angioectasia

**Figure 2:**
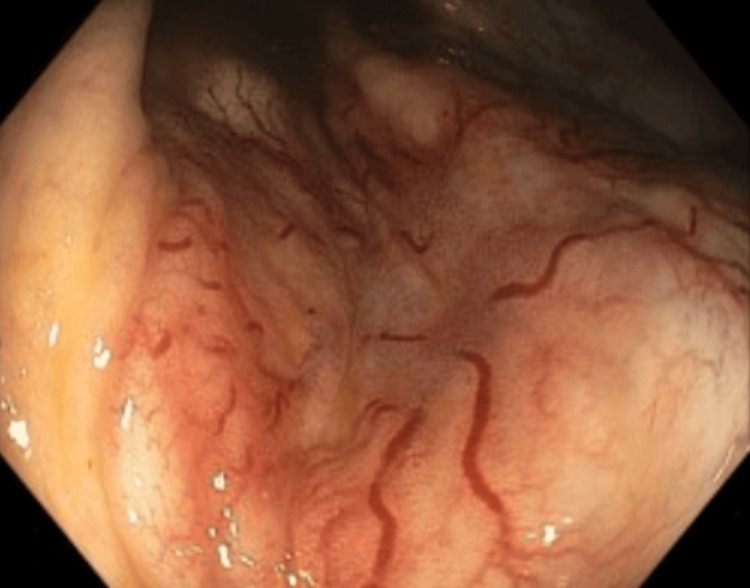
Colonoscopy demonstrating large atypical colonic angioectasia

Given the findings consistent with IDA, and after optimizing his heart failure status, he subsequently underwent an esophagogastroduodenoscopy (EGD) and colonoscopy to evaluate a potential source of blood loss. The EGD revealed mild erosive gastropathy without active bleeding or stigmata of recent hemorrhage. Biopsies were positive for *Helicobacter pylori*, and appropriate eradication therapy was initiated. Subsequently, the patient underwent a colonoscopy, which demonstrated multiple colonic polyps and a large, non-bleeding colonic angioectasia in the cecum, as seen in Figures 1, 2.

**Figure 3:**
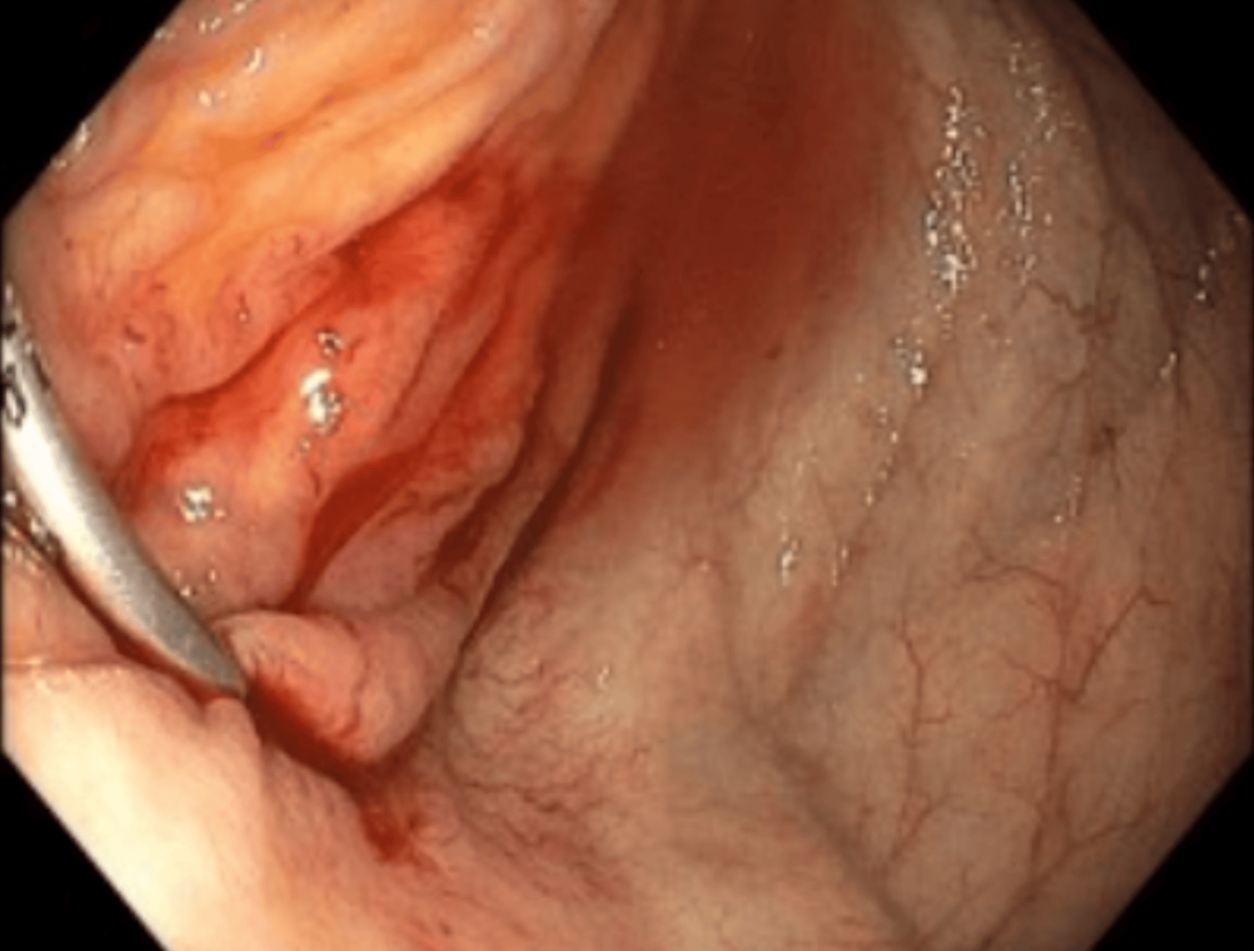
Colonoscopy demonstrating hemostasis achieved with clipping

Hemostasis was achieved by placing hemostatic clips at the site, as shown in Figure 3. Although active bleeding was not visualized during colonoscopy, the lesion was considered the most likely source of chronic blood loss given its size, location, and the patient's transfusion-requiring iron-deficiency anemia. Therefore, endoscopic therapy was performed to prevent further bleeding.

**Figure 4:**
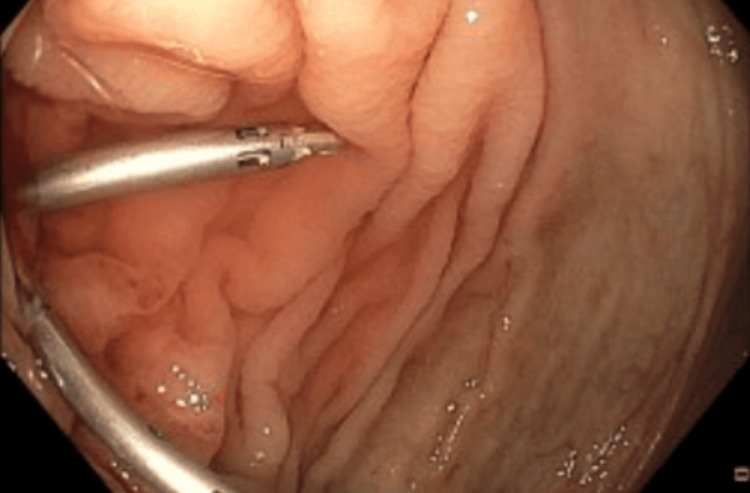
Colonoscopy demonstrating rectal varices

Additionally, rectal varices were noted, as seen in Figure 4, consistent with his history of cirrhosis and portal hypertension. Although rectal varices were present, there was no evidence of active bleeding or stigmata of recent hemorrhage, making them a less likely explanation for the patient's IDA compared with the large cecal vascular lesion. Several polyps, measuring approximately 10 mm, were found in the transverse, ascending, sigmoid, and descending colon, and polypectomy with clipping was performed.

The patient was advised to repeat the colonoscopy in one year for surveillance. He also started on carvedilol (Coreg) for the management of rectal varices in the setting of clinically significant portal hypertension. Following the procedure, he was observed for 24 hours; his hemoglobin remained stable, and he was subsequently discharged in stable condition.

## Discussion

Angioectasias are acquired vascular malformations that typically occur with advancing age. The development of colonic angioectasias is multifactorial, often linked to mild chronic venous obstruction and sustained mucosal hypoxia, which leads to increased expression of vascular endothelial growth factor (VEGF) [3]. Within the GI tract, angioectasias are most often in the large intestine, with the majority occurring on the right side of the colon (54-89%). Their prevalence ranges from 0.8% to 6.2% and is higher in patients with conditions such as aortic stenosis, von Willebrand disease (vWD), and chronic kidney disease. These lesions more frequently occur as multiple rather than solitary lesions, affecting 40-60% of cases [4]. Among healthy, asymptomatic individuals over 50 years of age, angioectasias have a prevalence of approximately 8% and account for 3-12% of cases of acute lower GI bleeding [5]. GI bleeding caused by angioectasia in patients with aortic stenosis is referred to as Heyde syndrome, named after Edward Heyde, who first identified this association in 1958 [6].

The exact mechanism behind angioectasia is not fully understood. Suggested explanations include chronic, intermittent low-grade venous obstruction that leads to dilated submucosal veins and abnormal arteriovenous connections. Other theories involve angiogenic factors and a link with aortic stenosis, where reduced von Willebrand factor, due to turbulent blood flow, may contribute to lesion formation [7,8]. 

Angioectasia can be diagnosed through colonoscopy, angiography, and CT scanning. Because angioectasia is omnipresent throughout the GI tract, a combination of studies with endoscopy may be necessary. While endoscopic forceps biopsy can show typical histologic features such as dilated, distorted, thin-walled vessels, it is generally discouraged due to its low diagnostic yield and the potential risk of causing bleeding [9]. The angiodysplastic lesions are usually small, flat, cherry-red, and typically under 10 mm in size [10]. 

Colonoscopy remains the preferred diagnostic modality and is highly effective in identifying colonic angioectasias [11]. Detection can be challenging due to factors such as small lesion size, location behind mucosal folds, or poor bowel preparation, especially during urgent procedures. Additionally, angioectasia bleeding is often slow, venous, and intermittent, which can limit the effectiveness of multiphase CT angiography and conventional angiography, as these imaging modalities only identify bleeding when rates exceed 0.3-0.5 mL/minute and 0.5-1.0 mL/minute, respectively. It also allows for simultaneous therapeutic intervention [12,13].

Radionuclide scanning, using either 99mTc-labeled autologous red blood cells or technetium sulfur colloid, is highly sensitive for detecting active bleeding. Red cell scintigraphy is preferred because its longer half-life allows imaging up to 24 hours, improving detection of intermittent bleeding. However, these scans may only localize a general bleeding area and cannot always pinpoint the precise site due to intestinal peristalsis [14]. 

For obscure small bowel bleeding due to angioectasia, capsule endoscopy or deep enteroscopy is recommended when initial evaluations are inconclusive. Intraoperative enteroscopy is employed as a final diagnostic option when endoscopic and radiologic methods cannot identify the source of bleeding. The procedure involves inserting an endoscope orally, rectally, or through an enterotomy during surgery, with a diagnostic yield of 60-88%. It is rarely performed due to potential complications, including perforation, tears of the serosa or mesentery, azotemia, and prolonged ileus [15,16].

Management of angioectasia depends on whether it is an incidental finding, nonbleeding in a patient with GI bleeding, or actively bleeding. Incidental angioectasia in asymptomatic patients without anemia generally does not require treatment, while nonbleeding lesions in patients with GI bleeding are often treated based on expert opinion. Actively bleeding angioectasia is managed similarly to other GI bleeds, with treatment guided by hemodynamic stability. Unstable patients require immediate fluid resuscitation, airway protection, intensive monitoring, and possible endoscopic, radiologic, or surgical intervention. In patients with acute overt bleeding, endoscopic evaluation is generally recommended within 24 hours. In patients with chronic IDA, the timing of evaluation is guided by clinical stability and the severity of anemia. [17]. 

When angioectasia is identified during endoscopy or colonoscopy, several treatment options are available. Argon plasma coagulation (APC) is the most commonly used method, delivering high-frequency energy via ionized gas; thorough bowel preparation is essential to prevent colonic gas explosions, and there is a higher risk of perforation in the upper GI tract [18,19]. Several studies and guideline-type reviews indicate that once a lesion, such as a colonic angioectasia, has been identified (most commonly in the cecum or right colon), endoscopic coagulation modalities, especially APC, should be used to ablate the abnormal vessels [20].

Electrocoagulation, using bipolar or heater probes, is better suited for the lower GI tract. Endoscopic clips and band ligation are both mechanical methods. Band ligation can be used on lesions in the stomach and small bowel. In our case, the colonic vascular lesion was notably larger than the typical flat or small angioectasias often encountered in the right colon. Given the size and the visible arterial component, we opted for endoscopic clipping to mechanically occlude the lesion and minimize the risk of immediate or delayed bleeding. Endoscopic clip placement has been described as an effective alternative in cases where the vascular lesion is large, pulsatile, or not amenable to APC due to the risk of transmural injury. Several reports and small series support this approach, noting that mechanical hemostasis using clips can provide durable control of bleeding and may reduce recurrence compared with thermal methods alone, especially when the feeding vessel is prominent or ulcerated [20]. Injection sclerotherapy involves injecting a sclerosant, such as ethanolamine or sodium tetradecyl sulfate, to obliterate angioectasias and other vascular lesions in the GI tract. Other endoscopic treatment options include ligation, resection, and photocoagulation [21,22].

First-line therapy for colonic angioectasias remains endoscopic ablation with APC or bipolar coagulation for smaller lesions. However, in cases of larger AVMs or nodular angioectasias with a defined feeding vessel, a combination approach, including clipping, injection therapy, or even band ligation, has been described to achieve definitive hemostasis [23].

Regarding medical management, angiogenesis inhibitors such as thalidomide and bevacizumab have been used to treat GI vascular malformations, including angioectasia [24]. In one clinical trial, thalidomide achieved a >50% reduction in GI bleeding in 71.6% of patients, compared to 3.7% in controls [25]. While effective in refractory or transfusion-dependent cases, thalidomide carries significant side effects, including teratogenicity, and must be used cautiously. Bevacizumab, a monoclonal antibody targeting VEGF, has also been reported to help in refractory cases [26], but limited evidence means it should be reserved as a last-resort therapy. Hormone therapy using estrogen, with or without progesterone, has been studied for chronic obscure GI bleeding. However, evidence, including randomized controlled trials, has not demonstrated any benefit in preventing angioectasia-related GI bleeding [27]. Octreotide has shown effectiveness in treating refractory GI bleeding due to angioectasia, according to case series and meta-analyses. It can be administered as twice-daily subcutaneous injections (50-100 mcg) or as a long-acting intramuscular form (octreotide-LAR (long-lasting release)) given monthly. Studies report a 73-76% response rate, reflected by reduced bleeding episodes, lower transfusion requirements, and improved hemoglobin levels. Therefore, octreotide may be considered for patients with refractory angioectasia-related bleeding [28-31].

When endoscopic management fails or the bleeding is recurrent, angiographic embolization or segmental colectomy may be required, particularly in hemodynamically unstable patients or those with extensive vascular malformations. In our case, mechanical clipping was successful in achieving hemostasis without recurrence on follow-up, underscoring its role as a safe and effective option for large colonic vascular lesions where thermal coagulation alone carries increased procedural risk. Angiography is typically used for patients with active bleeding who have not responded to other treatments, are poor surgical candidates, or require preoperative localization of bleeding. Therapeutic options include temporary embolization with absorbable gelatin sponges, local vasopressin infusion, or permanent embolization using micro-coils, particles, or glue [32]. Complications can include bowel ischemia or infarction, with initial hemostasis success rates of 95% for lower GI bleeding and 64-89% for upper GI bleeding. Rebleeding rates after lower GI embolization range from 22% to 48% [33].

Surgery is reserved for patients with severe bleeding requiring multiple transfusions when other measures fail. Rebleeding may occur due to incomplete resection or missed lesions. Preoperative localization using push or intraoperative enteroscopy, angiography, or endoscopic marking with clips or dye can improve surgical outcomes. In patients with aortic stenosis, aortic valve replacement may reduce angioectasia-related bleeding [34].

## Conclusions

This case describes an unusually large cecal angioectasia presenting as transfusion-dependent IDA without overt GI bleeding in a patient with multiple potential causes of anemia, including chronic kidney disease, cirrhosis, and heart failure. It emphasizes the necessity of a high index of suspicion for colonic angioectasia in elderly patients with unexplained IDA, even in the absence of active bleeding during evaluation. The case further illustrates that large vascular lesions can be a source of chronic occult blood loss and that mechanical clipping is a viable endoscopic treatment in selected cases. Thorough endoscopic evaluation for occult GI bleeding should be considered in similar patients, with therapy individualized according to lesion characteristics and patient comorbidities.
